# Prognostic and Predictive Significance of Selected Gene Mutations in Pancreatic and Intestinal Neuroendocrine Tumors

**DOI:** 10.3390/ijms27114874

**Published:** 2026-05-28

**Authors:** Jan Musiałkiewicz, Bartłomiej Budny, Aleksandra Anioła, Adam Maciejewski, Paweł Komarnicki, Joanna Maciejewska, Paweł Gut, Marek Ruchała

**Affiliations:** Department of Endocrinology, Metabolism and Internal Diseases, Poznan University of Medical Sciences, Przybyszewskiego 49, 60-355 Poznań, Poland; bbudny@ump.edu.pl (B.B.); aleksandra.aniola@usk.poznan.pl (A.A.); pawel.komarnicki@usk.poznan.pl (P.K.); joanna.sienkiewicz@usk.poznan.pl (J.M.); gutpawel@ump.edu.pl (P.G.); mruchala@ump.edu.pl (M.R.)

**Keywords:** neuroendocrine tumors, NETs, predictive, prognostic, mutations, biomarkers

## Abstract

Neuroendocrine tumors (NETs) constitute a heterogeneous and predominantly malignant group of neuroendocrine neoplasms that arise from endocrine cells dispersed throughout the body. Their clinical presentation, biological behavior, prognosis, and therapeutic management vary considerably depending on the primary tumor location and hormonal activity. Despite substantial progress in understanding the biology of NETs, identifying reliable molecular biomarkers for diagnosis, prognosis, and prediction of treatment response remains a major challenge. Increasing attention has therefore been devoted to the molecular characterization of NETs, with particular focus on recurrent genetic alterations that may contribute to tumor initiation and progression. In this review, we summarize current knowledge and recent findings referring to certain genes involved in the tumorigenesis of pancreatic and intestinal neuroendocrine tumors. We chose the genes based on data from the COSMIC (Catalogue of Somatic Mutations in Cancer) database, which compiles somatic mutations identified across numerous human cancers. We outline the biological functions of these changes and discuss their potential prognostic and predictive role as molecular markers. We also discuss their clinical relevance in both sporadic and familial forms of NETs, alongside their implications for future research and personalized management strategies.

## 1. Introduction

Neuroendocrine tumors (NETs) constitute a heterogeneous group of neuroendocrine neoplasms (NENs) with variable malignant potential that differentiate from neuroendocrine cells dispersed throughout the body. Historically, they were named carcinoids; however, this term has been replaced and is now mainly used for tumors in the bronchopulmonary and thymic locations [1]. The incidence of neuroendocrine tumors has been increasing in recent years and is estimated at approximately 5.86–9.39/100.000 population/year [2,3]. Although they are classified as rare diseases, they attract considerable attention from physicians of various specialties due to their complexity, heterogeneity, and diverse clinical presentation. The 2022 World Health Organization (WHO) criteria for NENs separate well-differentiated NETs from poorly differentiated neuroendocrine carcinomas (NECs). The degree of malignancy, or grade (G1, G2, and G3) of these tumors, depends on the Ki-67 index and the mitotic count [4]. The more common G1 and G2 NETs are characterized by slower growth and a less aggressive course than G3. However, they are often diagnosed at an advanced and metastatic stage, which impacts patients’ quality and length of life and necessitates long-term treatment.

From an embryological point of view, NETs can be categorized according to their origin from the foregut, midgut, or hindgut [5]. They are most commonly found in the gastrointestinal tract and pancreas, where they are known as gastroenteropancreatic NETs (GEP-NETs). The second largest group consists of pulmonary carcinoids [6]. NETs may have the ability to produce hormones, although the majority of them are hormonally inactive. The secretion of serotonin and other biogenic amines, characteristic of tumors originating from the intestine and lungs, leads to carcinoid syndrome (CS). CS is a set of symptoms such as abdominal pain, diarrhea, and facial flushing. It has a negative impact on the course of the disease and may lead to carcinoid heart disease, which should be actively screened for [7]. Pancreatic neuroendocrine tumors (panNETs) may also, in up to 20% of cases, produce specific hormones such as insulin, glucagon, vasoactive intestinal peptide, somatostatin or gastrin [8]. In these situations, the clinical presentation and symptoms will depend on the type of hormone released. The secretion of these hormones may lead to earlier symptom onset and thus an earlier diagnosis of NETs [9].

One of the greatest challenges in NETs is the lack of sensitive and specific markers, whether diagnostic, prognostic, or predictive [10]. Despite the passage of time, chromogranin A still remains one of the most commonly used biochemical markers. Its sensitivity and specificity are unsatisfactory, and its role is currently being reduced mainly to disease monitoring and the assessment of recurrence or progression [9,10,11]. The impact of others, such as pancreatic polypeptide, neuron-specific enolase, beta-human chorionic gonadotropin, or alpha-fetoprotein, is limited only to selected clinical situations [11,12,13,14]. Promising results in this area have been shown for visfatin, but studies on this marker require further exploration [15].

In NETs, liquid biopsy whole-exome and whole-genome sequencing studies based on the analysis of genetic alterations have also been investigated. A liquid biopsy refers to the analysis of a blood sample that carries tumor-derived information (circulating tumor cells, DNA and RNA) and can be obtained non-invasively compared with a conventional tissue biopsy. The best-known and most standardized tool is the NETest, which was reported to have very high sensitivity and specificity [16,17]. Its limitations, however, are its high cost and restricted availability, which prevent its wider dissemination. Moreover, the NETest has not received Food and Drug Administration (FDA) approval. Its clinical validity remains debated due to limited independent validation and the lack of full transparency of the proprietary algorithm. Despite that, data regarding genetic alterations contributing to the development of NETs are steadily increasing. Understanding these changes, analyzing them, and conducting prospective studies may, in the future, allow us to fill the gap associated with the limitations of biomarkers.

In recent years, intensive research has been ongoing to identify suitable prognostic and predictive markers in NETs. Particular attention has been directed toward genetic alterations, including those detectable through liquid biopsy, as well as mutation profiling in these tumors [18]. The aim of our article is to review the current existing knowledge and latest findings on selected gene alterations in the most commonly localized (pancreatic and intestinal) NETs, as well as their potential use as molecular markers. We would also like to discuss their prognostic and predictive value in clinical practice in both familial and sporadic NETs.

## 2. Literature Search Strategy

We conducted a structured literature search in PubMed to identify studies relevant to the topic. The search focused on well-differentiated neuroendocrine tumors (WD-NETs, WHO Grades 1–3) of the pancreas, small intestine, and large intestine, explicitly excluding poorly differentiated NECs due to their distinct clinical behavior. This was a narrative review with a systematic literature search strategy, aiming to provide a comprehensive description of genetic alterations in selected NET primary locations. In order to maintain a systematic approach to this narrative review, the search and screening processes were conducted in several phases.

### 2.1. Gene Selection

Key genes recurrently altered in NETs were identified through a preliminary analysis of genes contributing to NET tumorigenesis according to the COSMIC (Catalogue of Somatic Mutations In Cancer) database. The selection of genes was primarily based on their frequency of reporting in the COSMIC database. Priority was given to genes identified in both pancreatic and intestinal localization datasets. Chosen genes included: *MEN1*, *ATRX*, *DAXX*, *TSC2*, *TP53*, *ARID1A*, *VHL*, *PTEN, MTOR*, *CDKN1B*, *SMAD4*, *RB1*, *APC*, *CTNNB1*, and *NF1*. These genes formed the basis of the search query and were also evaluated for their prognostic and predictive utility.

### 2.2. Search Query

The PubMed search combined tumor terminology (taking into account multiple spelling versions and plural forms), NET primary locations, preselected genes, and biomarker-related terms using Boolean operators. The following search string was used:

(“neuroendocrine” OR NET OR NETs OR NEN OR NENs)
AND
(tumor OR tumors OR tumour OR tumours OR neoplasm OR neoplasms)
AND
(pancreas OR “small intestine” OR “large intestine” OR midgut)
AND
(*MEN1* OR *ATRX* OR *DAXX* OR *TSC2* OR *TP53* OR *ARID1A* OR *VHL* OR *PTEN* OR *MTOR* OR *CDKN1B* OR *SMAD4* OR *RB1* OR *APC* OR *CTNNB1* OR *NF1*)
AND
(biomarker OR biomarkers OR prognostic OR predictive OR marker OR markers)

### 2.3. Study Selection

**Figure 1:**
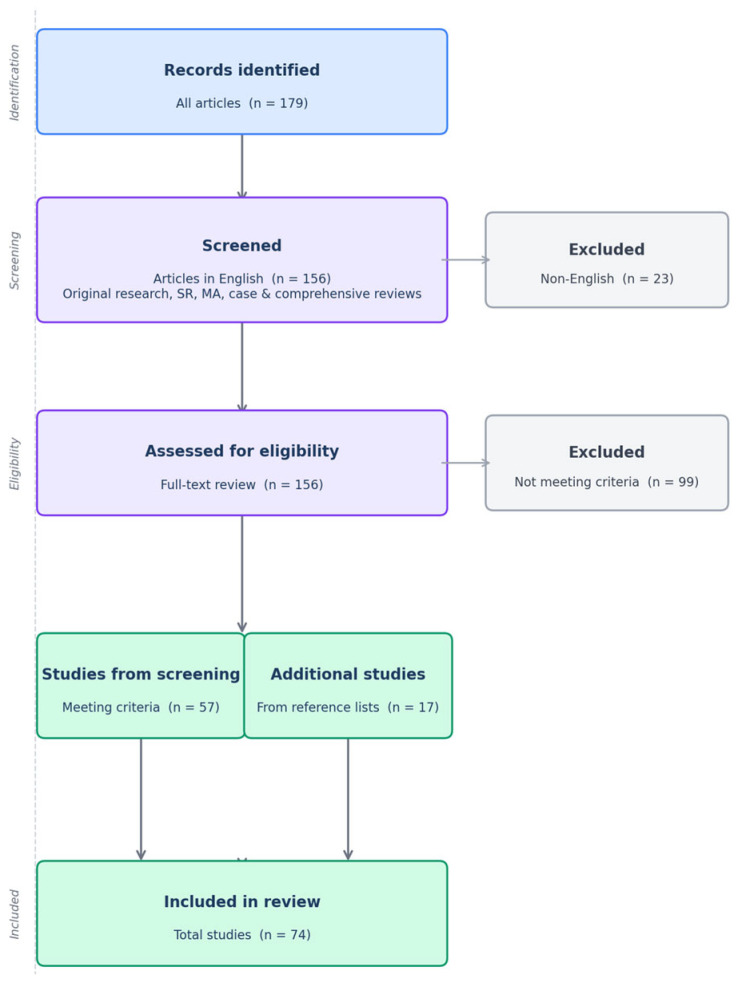
Flow chart illustrating study selection.

Eligible studies included original research articles, systematic reviews, meta-analyses, case reports, and comprehensive reviews. Letters to the editor, editorials, commentaries, conference abstracts, and book chapters were excluded. Titles and abstracts were screened independently by two reviewers (AA and JS) for their adherence to the inclusion criteria and relevance to the study topic. We included studies published between January 2010 and 15 October 2025. In cases of discrepancy, final decisions were made with input from PK and JM. Only articles in English were selected. After the completion of the study selection process, 57 articles were chosen to be included in this review, with 17 additional studies retrieved from the references of the source materials. The complete flow chart is presented in Figure 1.

## 3. Discussion

### 3.1. Pancreatic NETs

Pancreatic neuroendocrine tumors are characterized by a complex genetic landscape that is more diverse and more frequently altered than in NETs of the small or large intestine. Whole-genome analysis of NETs has revealed driver somatic mutations in recurrent genes that impact four major pathways: (1) chromatin remodeling, (2) DNA damage repair, (3) mTOR signaling activation, and (4) telomere maintenance [19,20]. Likewise, genes involved in cell cycle regulation, the Wnt signaling pathway, and tumor suppressors have also been identified [20]. An overview of the above-mentioned mechanisms and their related genes selected in our review is provided in Table 1.

The majority of panNETs occur sporadically, while others develop in the context of genetic syndromes such as multiple endocrine neoplasia type 1 (MEN1) and 4 (MEN4), tuberous sclerosis complex (TSC), neurofibromatosis type 1 (NF1), and von Hippel–Lindau disease (VHL) [21,22,23]. Those linked to genetic syndromes are generally regarded as less aggressive and tend to have a more favorable prognosis [8].

#### 3.1.1. MEN1

Multiple endocrine neoplasia type 1 (MEN1) syndrome is caused by an inherited or de novo germline autosomal dominant mutation in a tumor suppressor gene located on chromosome 11q13, which encodes a 610-amino-acid protein called menin [22]. The function of menin is associated with the regulation of cell growth, apoptosis, and DNA repair [24]. More than 1100 mutations have been detected across the entire coding region [25]. MEN1 syndrome shows high penetrance, and its clinical manifestations vary widely [26]. Patients diagnosed with MEN1 syndrome exhibit a significantly increased risk of developing multiple endocrine neoplasms at an early age, with over 90% affected by the age of 40 years [27]. Tumors most often occur as parathyroid gland adenomas (90%), followed by pancreatic or duodenal NETs (50%) and anterior pituitary adenomas (40%) [28]. PanNETs appear to be the most challenging and constitute the leading cause of death in patients with MEN1 (40–50%) [24,29].

In panNETs, sporadic mutations in the *MEN1* gene occur even more frequently, with their prevalence estimated at up to 44% [30]. Notably, these alterations appear to be restricted to panNETs and are absent in pancreatic NECs [31]. Considering the frequency of these mutations, it is justified to determine their impact on the disease course.

Chiloiro et al. compared the clinical, histological, and morphological aspects of panNETs in patients with and without MEN1 syndrome [32]. They found that the frequency of liver and lymph node metastases was higher in *MEN1* wild-type patients. Moreover, the mutation-positive group showed a more favorable prognosis, but this difference was not statistically significant. It can be partly explained by earlier diagnosis and improved screening due to active surveillance of these tumors in first-degree relatives.

These findings are in line with those of Gleeson et al., who demonstrated that patients harboring a *MEN1* variant developed pancreatic NETs of smaller size and were more frequently diagnosed at an early disease stage [33]. Other studies also emphasize a more favorable disease course and slower progression in patients with these mutations [19,21,24,27,34]. It should be noted, however, that within the *MEN1* group, prognostically unfavorable genetic factors may also occur. One such factor is a mutation in *c-MET*, a proto-oncogene encoding the c-MET tyrosine kinase receptor for hepatocyte growth factor. Ghosh et al. demonstrated that overexpression of *c-MET* is associated with a more aggressive disease course and poorer prognosis, similar to what is observed in other malignancies associated with mutations in this gene [35].

#### 3.1.2. VHL

Von Hippel–Lindau (VHL) syndrome is an inherited cancer predisposition disorder with autosomal dominant transmission, caused by a germline mutation in the *VHL* gene located on the short arm of chromosome 3 [36]. The *VHL* gene is responsible for regulating the cellular oxygen-sensing pathway by controlling the stability of hypoxia-inducible factors (HIFs), and its inactivation results in aberrant activation of genes involved in angiogenesis, cell proliferation, and metabolism [26]. Mutations in this gene lead to the development of clear cell tumors affecting multiple organs, including hemangioblastomas of the central nervous system and retina, renal cell carcinoma, pheochromocytomas, panNETs, and adrenal cortical adenomas [37]. In addition to germline mutations, the *VHL* gene is also frequently inactivated in up to 25% of sporadic panNETs, mostly by non-mutational mechanisms [30].

PanNETs occur in 10–17% of patients with VHL syndrome and are usually small, non-functioning (NF), well- or moderately differentiated tumors with high expression of somatostatin receptor analogs [34,38]. The majority are identified at a relatively young age, with a mean age at diagnosis of 26–38 years, and typically present as multifocal lesions distributed throughout the pancreas [27,38]. Malignant transformation of these tumors is relatively uncommon [24]. Compared with sporadic panNETs, VHL-associated tumors are far less often high-grade or metastatic and have better long-term outcomes [27,39]. However, it should be noted that several risk factors associated with panNET metastasis in VHL disease have been identified, including tumor diameter exceeding 3 cm, blood type 0, a tumor doubling time of less than 500 days, and missense mutations located in exon 3 of the *VHL* gene [36,38].

Considering the above, the presence of a *VHL* mutation should be regarded as a positive prognostic factor. This also applies to the majority of cases in which 18F-FDG PET scans show increased glucose uptake. This phenomenon may be explained not by high tumor grade but rather by the mechanism of *VHL* gene inactivation, which leads to accumulation of HIF protein, a pseudohypoxic state, and direct regulation of glucose transporter 1, responsible for cellular glucose uptake [40]. This phenomenon applies to both sporadic *VHL* mutations and VHL disease-related panNETs.

#### 3.1.3. DAXX and ATRX

Among sporadic panNETs, approximately 45% present inactivating somatic mutations in mutually exclusive tumor suppressor genes: *DAXX* (death-domain-associated protein, located on chromosome 6p21.3) or *ATRX* (alpha-thalassemia/mental retardation syndrome X-linked, located on chromosome Xq21.1). Both genes encode proteins involved in chromatin remodeling. Their mutations result in loss of expression of the corresponding protein, as assessed by immunohistochemistry (IHC) [21,34,41,42]. In the Hechtman study, the sensitivity of translating *DAXX* alterations into IHC was 95% [43]. The consequence of these mutations is the activation of the alternative lengthening of telomeres (ALT) mechanism, used by several types of malignancies, including panNETs. This mechanism allows neoplasms to maintain telomere length, supporting sustained tumor growth and cell proliferation [42,44]. Mutations in the *DAXX* and *ATRX* genes represent the second most common alterations in panNETs after *MEN1*. Similar to *MEN1* gene alterations, *DAXX* and *ATRX* mutations occur exclusively in panNETs and have not been reported in panNECs to date [31].

Based on the association between an activated ALT mechanism and *DAXX/ATRX* mutations, Luchini et al. suggested the determination of ALT as a potential prognostic biomarker and demonstrated that its presence was associated with poorer prognosis in panNETs [44]. These findings were supported by Marinoni et al., who showed a significant association between ALT activation and T stage and reported that tumors positive for ALT were characterized by significantly shorter relapse-free survival and reduced disease-specific survival (DSS) [45]. Similar conclusions were described in the study by McGovern et al., in which panNETs were assessed preoperatively using CT scans, and negative prognostic features were strongly correlated with ALT-positive status [46]. Of interest are also the studies by Pea and Hackeng, in which they demonstrated that ALT-positive patients have an increased risk of liver metastases in the panNET < 3 cm and insulinoma groups, respectively [47,48]. Moreover, Singhi et al. reported that patients with ALT-positive panNETs have a 40% 5-year disease-free survival (DFS), compared with 96% in ALT-negative patients [49].

In addition to its prognostic role, ALT status may also serve as a marker of pancreatic origin, supporting the diagnostic process in patients with neuroendocrine metastases from an unknown primary [44]. However, *DAXX* and *ATRX* mutations, together with their associated consequences, such as activation of the ALT mechanism, may also be observed, although less frequently, in extrapancreatic NETs [1,42,50].

The implications of ALT activation overlap with findings associated with mutations in the *DAXX* and *ATRX* genes. In the aforementioned study by Gleeson et al., the authors demonstrated that tumors harboring *DAXX* and/or *ATRX* mutations were larger at diagnosis, exhibited more malignant features, and were more frequently observed at advanced disease stages [33]. Similar conclusions were reached in many other studies [42,50,51,52,53]. However, in the study by Chen et al., in which loss of these proteins was correlated with a higher Ki-67 index and higher WHO grade of NETs, only *DAXX* loss reached statistical significance [50]. These correlations with more aggressive tumor potential also extend to functioning panNETs, such as glucagonomas, as demonstrated in the study by Mattiolo et al., in which *ATRX* or *DAXX* mutations were detected in the majority of cases [54].

Additional evidence for a worse prognosis in patients harboring *DAXX/ATRX* mutations comes from the study by Hong et al., where the presence of these mutations was associated with significantly shorter recurrence-free survival (RFS) [55]. This was also confirmed in the study by Hackeng, in which the combination of ALT-positive and/or *ATRX/DAXX*-negative NF-panNETs was correlated with markedly shorter RFS. This association was also observed in tumors < 2 cm [52]. Similarly, in extrapancreatic NETs, including those of the duodenum, appendix, and colon, overall survival (OS) was lower in patients who had lost *ATRX* or *DAXX* expression [20]. It should also be noted that an opposite correlation has been observed in patients with established metastatic panNETs, in whom loss of *DAXX/ATRX* was identified directly in metastases. This factor resulted in a better prognosis in several studies [20]. Taken together, the available evidence supports the recognition of *DAXX* and *ATRX* mutations and ALT activation (altered in primary origin) as independent negative prognostic factors.

#### 3.1.4. MTOR

The *MTOR* gene (the mammalian target of rapamycin), located on chromosome 1p36, encodes a serine–threonine kinase complex that serves as a central regulator of cell growth, proliferation, metabolism, and survival through the integration of nutrient and growth factor signaling. It becomes activated through phosphorylation, resulting in the formation of phosphorylated mTOR (p-mTOR) [56]. In panNETs, genomic alterations affecting components of the PI3K/AKT/mTOR pathway are observed in approximately 15% of cases and can be reflected by immunohistochemical staining of phosphorylated proteins of the pathway, contributing to its hyperactivation [34,56,57].

The expression of mTOR in panNETs has been evaluated as a potential prognostic marker. In the study by Lamberti et al., the prognostic significance of p-mTOR protein expression was assessed by calculating a quantitative score (QS) based on the percentage of positive cells and staining intensity during immunostaining evaluation. A high p-mTOR QS was shown to correlate with more advanced staging (IIIB–IV vs. I–II) and the presence of metastatic disease at diagnosis. Similarly, higher p-mTOR QS values were associated with disease relapse and shorter disease-free survival (DFS) [57]. In another study on panNETs conducted by Komori et al., elevated p-mTOR expression was likewise associated with more aggressive tumor behavior and adverse clinical characteristics, including larger tumor size, higher histological grade, and more advanced stage at diagnosis [56]. However, no significant association with DFS was observed, although the results showed a trend toward such a relationship (*p* < 0.1). The prognostic significance of *MTOR* has also been investigated in NETs of other locations, including the small intestine. In a study by Qian et al., mTOR expression was associated with a higher Ki-67 index, as well as shorter DFS and overall survival (OS) [58].

Clinically, aberrant mTOR signaling has predictive therapeutic relevance, as pharmacologic inhibition using mTOR inhibitors such as everolimus has demonstrated a significant improvement in progression-free survival (PFS) in patients with advanced pancreatic and extrapancreatic NETs [6]. In the study by Gelsomino et al., the expression of phosphorylated mTOR was examined during everolimus treatment and correlated with patient outcomes, as the mTOR-positive group had significantly better PFS and OS [59].

Overall, the presence of *MTOR* gene mutations appears to act as a positive predictive marker for response to everolimus therapy, while concurrently serving as a negative prognostic indicator for disease progression and clinical outcome.

#### 3.1.5. PTEN

Phosphatase and tensin homolog (*PTEN*) is a well-known tumor suppressor gene located on chromosome 10q23, a region commonly affected by somatic deletions in various tumors. It encodes a 403-amino-acid protein (PTEN) and plays an important role in controlling cell growth, apoptosis, cell adhesion, and cell migration [60]. In NETs, PTEN regulates the PI3K/Akt/mTOR pathway.

So far, its role as a prognostic marker in NETs has been well documented. Krausch et al. found in their research that loss of PTEN correlates with tumor progression and is associated with poorer outcomes and survival [35]. This is in line with the study by Missiaglia et al., in which reduced PTEN expression was linked to a more aggressive tumor phenotype. In addition, they demonstrated that low cytoplasmic PTEN expression was associated with a shorter time to disease progression and decreased disease-free survival [61]. Another observation was made by Estrella et al., confirming the significant negative prognostic role of *PTEN* alterations in NETs [62].

#### 3.1.6. TSC2

Tuberous sclerosis complex gene 2 (*TSC2*) is a tumor suppressor gene encoding tuberin and located on chromosome 16p13.3. Its germline mutation, together with the *TSC1* gene (encoding hamartin), results in the development of tuberous sclerosis complex, a rare inherited autosomal dominant syndrome. The molecular pathogenesis of the disease is driven by disruption of the normal activity of the hamartin–tuberin complex, leading to dysregulation of the mTOR pathway and resulting in unregulated cell proliferation and tumorigenesis [63]. The phenotypic manifestations of this disorder encompass the development of multiple hamartomatous lesions, a spectrum of neurological abnormalities—most prominently epilepsy and intellectual disability—and characteristic cutaneous features, including hypomelanotic macules, facial angiofibromas, shagreen patches, and ungual fibromas [27].

Existing case reports on *TSC2* as a molecular prognostic marker in NETs suggest that some younger patients may remain asymptomatic. These observations may be influenced by more intensive screening in individuals with tuberous sclerosis, similar to MEN1 syndrome [63,64]. In contrast to these reports, stronger evidence comes from the study by Gleeson et al., in which alterations in *TSC2* were linked to increased disease progression and worse overall survival (OS) [33]. Similar conclusions were reported by Missiaglia et al., who demonstrated that patients with reduced TSC2 expression showed poorer outcomes, including shorter OS, progression-free survival (PFS), and disease-free survival (DFS) [61]. Considering these findings, mutations in the *TSC2* gene should be regarded as a negative prognostic factor in patients with NETs.

#### 3.1.7. NF1

Neurofibromatosis type 1 (NF1), previously known as von Recklinghausen disease, is an inherited autosomal dominant condition resulting from pathogenic variants in the *NF1* gene. It encodes the tumor suppressor protein neurofibromin and is located on chromosome region 17q11.2 [27,30]. Inactivation of the *NF1* gene leads to constitutive upregulation of RAS signaling pathways, promoting the development of hallmark clinical and neoplastic features of the syndrome, including neurofibromas, café-au-lait spots, Lisch nodules, intertriginous freckling, and optic pathway gliomas [27,30].

In patients with NF1, neuroendocrine tumors can develop in approximately 10% of cases and are almost exclusively duodenal somatostatinomas of the periampullary region and, more rarely, nonfunctional panNETs [24,27,30,65]. Malignant forms occur in about 30% of cases [24]. The presence of sporadic *NF1* mutations in NETs is occasional, and there are currently insufficient data to determine their clinical significance [30]. According to the cited studies, duodenal and pancreatic somatostatinomas associated with NF1 differ from their sporadic pancreatic counterparts in multiple respects: syndromic tumors are identified at a smaller size, rarely induce a clinical hypersecretory state, and have a lower incidence of metastatic disease at the time of diagnosis [27]. To sum up, the presence of an inherited *NF1* gene mutation should be considered a positive prognostic marker, indicative of a more favorable clinical outcome and disease course.

#### 3.1.8. RB1

The *RB1* (retinoblastoma 1) gene is located on chromosome 13q14 and encodes the tumor suppressor retinoblastoma protein involved in cell cycle regulation. Inactivation of *RB1* through loss or mutation impairs this checkpoint mechanism, promoting uncontrolled cell division and contributing to the development of retinoblastoma as well as numerous other malignancies, including NENs. In NETs, *RB1* gene mutations are considered rare and are mainly observed in NET G3 metastases rather than in primary tumors [65]. Their presence is therefore indicative of advanced, disseminated disease. Conversely, in NECs, the prevalence of somatic mutations is high and estimated to be up to 74% of cases [21,34,37]. This may be a valuable supportive tool in the differential diagnosis of these types of NENs [1,66,67].

According to our review, there are no data on the predictive value of *RB1* in NETs. However, it is worth noting that in the subgroup of panNECs, there are reports indicating that loss of Rb protein expression associated with *RB1* gene mutations represents a positive predictive factor for response to first-line platinum-based chemotherapy, as the response rate to this treatment is higher than in the group without Rb loss.

On the other hand, this group is characterized by shorter OS, which confirms a more aggressive disease course [68]. Similar observations were made by Hijioka et al. [69]. Therefore, *RB1* gene mutations should be considered a negative prognostic factor (for NETs and panNECs) and a positive predictive factor (for platinum-based chemotherapy in panNECs).

#### 3.1.9. TP53

*TP53* is a tumor suppressor gene located on chromosome 17p13.1 that encodes the p53 protein, a key regulator of cell cycle arrest, DNA repair, senescence, and apoptosis in response to cellular stress. *TP53* mutations lead to abnormal p53 staining on immunohistochemistry (for example, in 100% of small cell and 90% of large cell neuroendocrine carcinomas) [21,34,37,67]. In the context of NENs, *TP53* alterations are rare in well-differentiated panNETs but occur frequently in poorly differentiated panNECs [1,66,68,70]. Similar to the *RB1* gene, the presence of *TP53* mutations should guide the diagnosis toward NEC [1,66,71].

There are numerous reports describing the impact of *TP53* mutations on the course of disease in panNETs. Ail et al. showed in their research that a mutated p53 pattern in patients with gastroenteropancreatic neuroendocrine neoplasms was associated with worse OS and DFS, irrespective of tumor grade [72]. Comparable conclusions have been reported by others [33]. Moreover, Kasajima et al. demonstrated an association between sudden and severe deterioration in all NET G3 patients’ condition and immunohistochemical overexpression of p53, accompanied by a corresponding mutation in the *TP53* gene [73]. Additional characteristics of *TP53* are presented in the intestinal NETs section.

#### 3.1.10. SMAD4

The mothers against decapentaplegic homolog 4 (*SMAD4*) gene encodes a key transcriptional mediator of the TGF-β signaling pathway and acts as a tumor suppressor by regulating cell proliferation, differentiation, and apoptosis. The gene is located on chromosome 18q21, and its alterations result in reduced or absent SMAD4 protein expression.

*SMAD4* alterations in pancreatic malignancies are primarily associated with pancreatic adenocarcinoma. Some studies indicate that *SMAD4* mutations are infrequently observed in pancreatic NECs and do not appear to play a significant role in these tumors [68,74]. However, these data are inconclusive, as conflicting evidence has been reported in other studies [31,75].

In gastrointestinal NETs, loss or inactivation of *SMAD4* is uncommon but has been associated with tumor progression, aggressive behavior, and poorer prognosis [76]. Further evidence supporting this hypothesis from the Martin et al. report indicates that *SMAD4* mutations are clinically significant and likely contribute to high-grade transformation in WD-panNETs [77].

#### 3.1.11. ARID1A

*ARID1A* (AT-rich interactive domain-containing protein 1A) is a tumor suppressor gene encoding a core subunit of the SWI/SNF (switch/sucrose nonfermentable) ATP-dependent chromatin-remodeling complex and plays a fundamental role in the regulation of chromatin accessibility, transcriptional programs, cell cycle progression, and DNA damage response. Recurrent loss-of-function alterations in *ARID1A* have been documented across a broad spectrum of human malignancies, including gastrointestinal, ovarian, and colorectal cancers and panNETs. The gene is localized to chromosome 1p36.11, and its inactivating mutations are associated with loss of the corresponding protein [51,78].

In the Han et al. study, *ARID1A* transcript levels were markedly downregulated in NF-panNETs compared with those in the adjacent normal pancreas. Among panNETs, two subgroups were identified based on ARID1A expression levels: a high-expression group and a low-expression group. Patients with reduced ARID1A expression more frequently exhibited regional lymph node metastases, larger tumor size, higher Ki-67 indices, and higher tumor grade [78]. These findings are consistent with the results reported by Roy et al., in which, among the evaluated biomarkers, loss of *ARID1A* was associated with more aggressive features of pancreatic neuroendocrine tumors and the presence of distant metastases. Moreover, patients in this subgroup exhibited significantly shorter DFS and DSS, underscoring the negative prognostic impact of *ARID1A* loss [51].

### 3.2. Intestinal NETs

The molecular landscape of intestinal NETs is characterized predominantly by chromosomal alterations and epigenetic changes, including DNA methylation, rather than by single somatic mutations [79,80,81]. Loss of chromosome 18 is considered the most frequent molecular event, occurring in approximately 63–82% of small intestinal NETs (siNETs), and is thought to be an early event in tumor development [80,82,83,84]. The overall somatic mutation burden is consistently reported to be noticeably lower than in panNETs. Depending on the cohort and methodology, in siNETs, driver mutations are detected in up to 34.6% of cases and are mostly located in tumor suppressor genes (*CDKN1B*, *TP53*) or, less frequently, in proto-oncogenes (*KRAS, NRAS, MET*) [82,85]. However, these alterations are typically non-recurrent and occur at lower frequencies. Driver mutations are detected more frequently in patients with a higher Ki-67 index [82]. Among colorectal NENs, the largest proportion consists of NECs, which are frequently characterized by mutations in *APC, KRAS, BRAF*, and *TP53* and often exhibit microsatellite instability [5].

#### 3.2.1. CDKN1B

The cyclin-dependent kinase inhibitor 1B gene (*CDKN1B*) is a tumor suppressor gene located on chromosome 12p13. The *CDKN1B* gene encodes the p27 protein, which is a key regulator of cell cycle progression [86]. Loss-of-function alterations or reduced expression of p27 contribute to dysregulated cellular proliferation and are considered early events in tumorigenesis [86]. Germline mutations in the *CDKN1B* gene are associated with MEN4 syndrome, a phenotype characterized by tumors affecting the parathyroid glands, pituitary, and pancreas, closely resembling the clinical presentation observed in MEN1 [21,86]. *CDKN1B* alterations can act as driver events in about 7–20% of sporadic siNETs [80,82,85,86,87]. Therefore, it is assumed to be the main somatic mutation observed in this group of NETs.

Data on the prognostic/predictive value of *CDKN1B* in intestinal NETs are very limited, with most studies focusing on its pathogenetic role. According to Crona et al., there is no clear agreement on the influence of these mutations on clinical characteristics and survival [86]. Similar conclusions were reached by Simbolo et al. in their study [85]. This is also partially supported by the study by Elias et al., who stated that the presence of *CDKN1B* gene mutations does not appear to be essential for metastasis formation [88]. On the other hand, Scarpa underlines that DFS analysis showed a progressively poorer prognosis in patients harboring *CDKN1B* alterations, although the results were mainly related to copy number variations (CNVs) rather than somatic mutations [79]. In summary, *CDKN1B* gene mutations appear to have unclear prognostic and predictive relevance so far.

#### 3.2.2. APC

The *APC* (adenomatous polyposis coli) gene is a tumor suppressor gene located on chromosome 5q21–q22 [89]. It encodes a multifunctional APC protein that negatively regulates the Wnt/β-catenin signaling pathway, controlling cell proliferation, differentiation, and genomic stability. The *APC* gene is well known for its role in familial and sporadic colon cancer, with a reported frequency of approximately 70% [89]. In less common cases, it has also been detected in tumors arising from the liver, stomach, and lung (notably squamous cell carcinoma and small cell carcinoma), as well as in breast cancer and cerebellar medulloblastoma [89].

One of the attempts to investigate the occurrence and impact of APC alterations in intestinal NETs was conducted by Bottarelli et al., who investigated 30 siNETs and reported that 23% harbored *APC* mutations and 15% displayed loss of one gene copy [89]. In a study by Simbolo et al., *APC* alterations were found in 7.7% of cases [85]. However, no clear correlation with clinical course and prognosis has been found so far [85,89]. Due to the limited availability of existing data, the prognostic and predictive significance of APC alterations in NETs remains largely undefined.

#### 3.2.3. CTNNB1

The *CTNNB1* (catenin beta 1) gene, located on chromosome 3p22.1, encodes β-catenin, which plays a crucial role in the canonical Wnt signaling pathway. Activating mutations in *CTNNB1* lead to stabilization and nuclear accumulation of β-catenin, promoting transcription of Wnt target genes involved in proliferation and tumor progression. It is most frequently associated with colorectal cancer but is also detected in other malignancies.

In our review, only a single study showed that in intestinal NETs, CTNNB1 methylation was increased in metastases [79]. None of the other studies specifically investigated mutations affecting this gene. Similar to the APC gene, there is currently no evidence regarding the prognostic or predictive significance of *CTNNB1* alterations in intestinal NETs.

#### 3.2.4. TP53

The introduction and molecular characteristics of the *TP53* gene have been presented in Section 3.1.9.

In intestinal neuroendocrine NENs, *TP53* mutations, similar to those in pancreatic NENs, are markedly more frequent in NECs than in NETs and may aid in distinguishing between these subgroups [71]. Data regarding the prognostic role of *TP53* in intestinal NETs remain limited; however, they appear consistent with observations in pancreatic NETs. In both studies by Ail et al. and Cubiella et al., an immunohistochemical p53 expression pattern indicative of *TP53* mutation, as well as mutation status assessed directly, was associated with poorer prognosis and reduced overall survival [71,72]. Therefore, *TP53* mutation is considered a negative prognostic factor in intestinal NETs, similar to the pancreatic group.

### 3.3. Conclusions and Future Directions

For many years, numerous attempts have been made to identify reliable biomarkers for NETs. Recent advances in genetics have improved our understanding of the mechanisms underlying tumorigenesis. In addition, minimally invasive approaches, such as liquid biopsy, now allow targeted profiling of these tumors, providing valuable insights into their biology. Genetic profiling may play a pivotal role in early diagnosis, prognostic assessment, detection of recurrence, and guiding individualized therapeutic strategies, particularly through the identification of novel molecular targets.

Our review highlights that, despite progress in understanding the prognostic value of genetic alterations in panNETs, their application as predictive biomarkers remains limited. In intestinal NETs, which exhibit markedly different tumorigenic mechanisms, available data are too limited to draw firm conclusions. Significant gaps in this research area persist, underscoring the need for further studies to fully realize their clinical potential. A detailed summary presenting the prognostic and predictive relevance of individual gene mutations is provided below in Table 2.

To conclude, gene mutations as molecular markers have diverse applications, including diagnostic differentiation between NETs and NECs, identification of unknown primary tumor sites, stratification of patients at higher risk of progression and poorer prognosis, and selection of appropriate therapies. Collectively, these applications contribute to a personalized decision-making process, which is expected to become increasingly valuable in modern medicine. However, clinical implementation of these biomarkers remains constrained by several significant limitations. One of them is the restricted availability of advanced molecular diagnostic techniques, particularly in low-resource healthcare centers. An additional challenge is the lack of standardized detection methods and insufficient clinical validation of many potential biomarkers in large, multicenter studies. Moreover, the heterogeneity of NETs within locations and the biological differences between locations further complicate the interpretation of results. Therefore, further studies are required to evaluate the actual clinical utility of these mutations (such as a risk stratification model or diagnostic workflow) and to facilitate their integration into current diagnostic and therapeutic algorithms. Additionally, there is a need for further investigation of emerging and less-studied biomarkers, as this research gap still exists.

### 3.4. Review Limitations

Our review focuses primarily on somatic mutations in NETs and does not consider epigenetics, transcriptomics, or other multi-omics approaches. Moreover, some evidence supporting the significance of specific genes comes from limited data, such as small patient cohorts or isolated case reports, which may reduce their clinical relevance. Finally, we searched the literature using a single database (PubMed only), which may introduce a potential risk of selection bias.

## Figures and Tables

**Table 1:** Gene mutations and their regulatory mechanisms in neuroendocrine tumors.

Mechanism	Gene Mutated	Localization
Chromatin remodeling	*MEN1*	Pancreas, small intestine
	*ARID1A*	Pancreas
mTOR signaling activation	*MTOR*	Pancreas, small intestine
	*NF1*	Pancreas, small intestine
	*PTEN*	Pancreas
	*TSC2*	Pancreas
Tumor suppressor	*TP53*	Pancreas, large intestine
	*RB1*	Pancreas, large intestine
	*VHL*	Pancreas
	*SMAD4*	Pancreas
	*CDKN1B*	Small intestine
Wnt signaling	*APC*	Small and large intestine
	*CTNNB1*	Small intestine
Telomere maintenance (ALT pathway)	*DAXX*	Pancreas
	*ATRX*	Pancreas

**Table 2:** Summary of the prognostic and predictive relevance of individual genes in NETs according to tumor location.

Gene	Tumor Location	Relevance	Mutation Effect	References
*MEN1*	Pancreas	Prognostic	Positive	Challis et al. 2019 [19] Reid et al. 2014 [21] Mauriello et al. 2015 [24] Guilmette et al. 2019 [27] Chiloiro et al. 2018 [32] Gleeson et al. 2017 [33] Reid et al. 2014 [34]
*ATRX*	Pancreas	Prognostic	Negative	Luchini et al. 2021 [44] Rindi et al. 2022 [1] Marinoni et al. 2014 [45] McGovern et al. 2018 [46] Singhi et al. 2017 [49] Hong et al. 2020 [55] Hackeng et al. 2022 [52] Mattiolo et al. 2024 [54] Gleeson et al. 2017 [33] Mastrosimini et al. 2023 [53] Heaphy & Singhi 2022 [42]
*DAXX*	Pancreas	Prognostic	Negative	Luchini et al. 2021 [44] Rindi et al. 2022 [1] Marinoni et al. 2014 [45] McGovern et al. 2018 [46] Singhi et al. 2017 [49] Hong et al. 2020 [55] Hackeng et al. 2022 [52] Mattiolo et al. 2024 [54] Chen et al. 2013 [50] Gleeson et al. 2017 [33] Mastrosimini et al. 2023 [53] Heaphy & Singhi 2022 [42]
*TSC2*	Pancreas	Prognostic	Negative	Missiaglia et al. 2010 [61] Gleeson et al. 2017 [33]
*TP53*	Pancreas, small and large intestine	Prognostic	Negative	Cubiella et al. 2024 [71] Ail & Paulose 2025 [72] Kasajima et al. 2024 [73] Gleeson et al. 2017 [33]
*ARID1A*	Pancreas	Prognostic	Negative	Han et al. 2020 [78] Roy et al. 2018 [51]
*VHL*	Pancreas	Prognostic	Positive	Guilmette & Nosé 2019 [27] Laks et al. 2022 [39] Reid et al. 2014 [34] Penitenti et al. 2021 [38]
*PTEN*	Pancreas	Prognostic	Negative	Krausch et al. 2011 [60] Missiaglia et al. 2010 [61] Estrella et al. 2014 [62]
*MTOR*	Pancreas, small intestine	Prognostic	Negative	Lamberti et al. 2017 [57] Komori et al. 2014 [56] Qian et al. 2013 [58]
		Predictive	Positive	Gelsomino et al. 2020 [59]
*CDKN1B*	Pancreas, small intestine	Unclear	Unclear	Simbolo et al. 2018 [85] Crona et al. 2015 [86] Elias et al. 2021 [88]
*SMAD4*	Pancreas, small and large intestine	Prognostic	Negative	Martin et al. 2018 [77] Roland et al. 2017 [76]
*RB1*	Pancreas, large intestine	Prognostic	Negative	Konukiewitz et al. 2017 [68]
		Predictive	Positive	Hijioka et al. 2017 [69]
*APC*	Small and large intestine	No evidence	No evidence	Simbolo et al. 2018 [85] Bottarelli et al. 2013 [89]
*CTNNB1*	Small intestine	No evidence	No evidence	Scarpa 2019 [79]
*NF1*	Pancreas, small intestine	Prognostic	Positive	Guilmette et al. 2019 [27]

## Data Availability

No new data were created or analyzed in this study. Data sharing is not applicable to this article.

## Abbreviations

The following abbreviations are used in this manuscript:

NET	Neuroendocrine tumor
NEN	Neuroendocrine neoplasm
WHO	World Health Organization
NEC	Neuroendocrine carcinoma
GEP-NETs	Gastroenteropancreatic NETs
CS	Carcinoid syndrome
PanNETs	Pancreatic neuroendocrine tumors
WD	Well-differentiated
COSMIC	Catalogue of Somatic Mutations In Cancer
MEN	Multiple endocrine neoplasia
TSC	Tuberous sclerosis complex
NF1	Neurofibromatosis type 1
VHL	Von Hippel–Lindau
HIF	Hypoxia-inducible factor
NF	Non-functioning
18F-FDG PET	Positron emission tomography using fluorine-18 labeled glucose
DAXX	Death-domain associated protein
ATRX	Alpha-thalassemia/mental retardation syndrome X-linked
IHC	Immunohistochemistry
ALT	Alternative lengthening of telomeres
RFS	Recurrence-free survival
DFS	Disease-free survival
DSS	Disease-specific survival
OS	Overall survival
mTOR	mammalian target of rapamycin
QS	Quantitative score
PFS	Progression-free survival
PTEN	Phosphatase and tensin homolog
SMAD4	Mothers against decapentaplegic homolog 4
ARID1A	AT-rich interactive domain-containing protein 1A
SWI/SNF	Switch/sucrose nonfermentable
SiNETs	Small intestine neuroendocrine tumors
CDKN1B	Cyclin-dependent kinase inhibitor 1B
APC	Adenomatous polyposis coli
CTNNB1	Catenin beta 1
